# SPETS: Secure and Privacy-Preserving Energy Trading System in Microgrid

**DOI:** 10.3390/s21238121

**Published:** 2021-12-04

**Authors:** Ahmed Samy, Haining Yu, Hongli Zhang, Guangyao Zhang

**Affiliations:** 1 School of Computer Science and Technology, Harbin Institute of Technology, Harbin 150001, China; ahmed.samy@hit.edu.cn (A.S.); yuhaining@hit.edu.cn (H.Y.); 2 Faculty of Computers and Information, Menoufia University, Shebin El-Kom 32511, Egypt; 3 Heilongjiang Branch of the National Internet Emergency Response Center, Harbin 150001, China; hello20080808@139.com

**Keywords:** peer-to-peer energy trading, blockchain, privacy, security, performance benchmarking

## Abstract

Recently, the development of distributed renewable energy resources, smart devices, and smart grids empowers the emergence of peer-to-peer energy trading via local energy markets. However, due to security and privacy concerns in energy trading, sensitive information of energy traders could be leaked to an adversary. In addition, malicious users could perform attacks against the energy market, such as collusion, double spending, and repudiation attacks. Moreover, network attacks could be executed by external attackers against energy networks, such as eavesdropping, data spoofing, and tampering attacks. To tackle the abovementioned attacks, we propose a secure and privacy-preserving energy trading system (SPETS). First, a permissioned energy blockchain is presented to perform secure energy transactions between energy sellers and buyers. Second, a discrete-time double auction is proposed for energy allocation and pricing. Third, the concept of reputation scores is adopted to guarantee market reliability and trust. The proposed energy system is implemented using Hyperledger Fabric (HF) where the chaincode is utilized to control the energy market. Theoretical analysis proves that SPETS is resilient to several security attacks. Simulation results demonstrate the increase in sellers’ and buyers’ welfare by approximately 76.5% and 26%, respectively. The proposed system ensures trustfulness and guarantees efficient energy allocation. The benchmark analysis proves that SPETS consumes few resources in terms of memory and disk usage, CPU, and network utilization.

## 1. Introduction

Traditional power grids rely heavily on fossil fuels to generate high power through large centralized power plants [1]. The use of fossil fuels produces carbon emissions which are directly linked to global climate change [2]. With the increasing demand for green energy, many countries opted to use renewable energy to satisfy future energy demand, reduce air pollution, and overcome the challenges of traditional power grids [3]. In the third quarter of 2020, 3.8 gigawatts (GW) of solar photovoltaics (PVs) have been installed to reach 88.9 GW of total installed capacity, enough to power 16.4 million houses in the U.S. [4]. The advent of renewable energy resources, smart homes, and smart grids provides potential for individuals to generate energy to satisfy energy demands, while surplus energy could be sold to neighbors to satisfy their local needs [5], whereby peer-to-peer (P2P) energy trading has witnessed surpassing growth over the past few years [6]. Many studies have concluded that the use of renewable energy contributes to countries’ economic progress [3,7]. Microgrid (MG) is a local energy grid with control capability which can operate with the traditional power grid in connected or isolated mode. MG integrates distributed energy resources (DER) with storage devices and flexible loads to comprise low voltage distribution systems and facilitate energy sharing [8]. With the distributed locations of renewable energy resources in MG, it allows energy traders to establish P2P energy transactions where residents can generate, store, and trade energy in a local energy market without the need for a third party [9]. P2P energy trading relies on a bi-directional network communication which makes the system vulnerable to several attacks [10]. Thus, energy trading brings security and privacy concerns to energy traders, such as private data leakage, data breaches, double spending, collusion, distributed denial of service (DDoS), and man in the middle (MITM) attacks.

Blockchain is an emerging and fast-developing technology that has received considerable attention in industry and academia. In contrast with centralized applications, blockchain enables applications to run in a decentralized fashion without the need for a third-party or intermediary [11,12]. Thus, considering the P2P energy trading properties, blockchain has profound implications in future energy transaction networks [13,14]. It provides a platform for linking participants, providing market access, enforcing market rules, and monitoring market operations. Recently, numerous approaches have been proposed to enable P2P energy trading in which blockchain technology is adopted to guarantee security and privacy. A consortium blockchain is utilized to address security challenges in P2P energy trading between individuals in [2,15] and between hybrid electric vehicles in [16]. Blockchain is used to manage crowd-sourced energy trading operations in [17] to enable P2P energy trading between individual prosumers and/or the utility. A framework was proposed in [18] to provide automated demand response energy transactions in energy local networks with decentralized scheduling utilizing blockchain technology.

Despite, the presence of several blockchain-based P2P energy trading frameworks, there are many challenges that are not sufficiently addressed. For example, the sensitive information of users, such as name, ID, location, bidding price, energy quantity, etc., could be extracted from energy transactions stored on the blockchain ledger. Moreover, a malicious user could submit insufficient energy quantity in the energy transaction, a legitimate user may cooperate with other users to maximize their profits to conduct a collision attack, and energy reads generated from the smart meters (SMs) could be intercepted and altered. Most of the existing papers considering P2P energy trading do not present a complete system design of energy trading in terms of market design and control, energy allocation and pricing, privacy consideration, and attacks mitigation. Further, researchers did not provide a performance analysis of their energy trading systems. These concerns motivate our work to address the security and privacy issues, market design problems, and performance evaluation issues.

In this context, a comprehensive HF-based energy trading system is proposed to address the challenges in the existing energy trading systems. A detailed market design is presented to receive, validate, and process requests from energy sellers/buyers. An efficient energy allocation and pricing are employed using the discrete-time double auction mechanism in which the sell/buy activity of participants is hidden from unauthorized access. The market stability and reliability are achieved using the market reputation score method. Moreover, the proposed system design increased the welfare of energy buyers and sellers. An extensive performance analysis is executed to verify the ability of HF to design efficient P2P energy trading in a scalable and high-response manner.

### Contributions

The main objectives of this work are investigating the potentials of efficiently cooperating HF in P2P energy trading and leveraging the capabilities of HF to design high performance, secure, and privacy-preserving P2P energy trading that is performed in situations such as grid-to-vehicle, vehicle-to-grid, and grid-to-grid. However, this paper focuses on P2P energy trading between individuals in a microgrid ecosystem. The contributions of this paper are summarized as follows:

- It proposes a decentralized energy trading system that can be effectively integrated with HF. The proposed system enables authorized users to trade energy in a secure, reliable, and privacy-preserving manner without a third-party.
- It explores the capability of HF as a management information system to immutably store transactions, ensures secure energy transactions, and protect the participant’s sensitive information, employing channels and private data collections (PDC).
- It proposes a lightweight and efficient discrete-time double auction with an average mechanism to allocate energy and calculates energy price. A market reputation score method is adopted to guarantee reliability and trust.
- It provides theoretical security analysis and quantitative performance analysis to prove the effectiveness of our proposed system and how it resists against several security attacks.
- It provides a benchmark study on the designed HF energy network using the Hyperledger Caliper tool (V0.4.0) to measure transaction latency, throughput, resource consumption, and network utilization.

This paper is organized in the following order. Section 2 reviews the background and highlights the related work to make a comparison with our proposed system. Section 3 provides a clear threat model and well-defined design objectives of the proposed SPETS. The main components and problem formulation of the proposed energy system are presented in Section 4. Section 5 shows the working mechanism of SPETS including energy market details, allocation, and reputation scores mechanisms. Furthermore, it represents the energy trading procedures that are carried out by energy traders. Section 6 shows the experimental methodology and Section 7 discusses the experiment results and benchmark analysis. Section 8 presents the conclusion and future work. Notations and acronyms are summarized in the Appendix A.

## 2. Background and Related Work

Due to the lack of adequate sources of energy generation, MG suffers from difficulties in fulfilling the energy demand. Consequently, a P2P energy trading is proposed to enable a person to sell surplus energy to another person who has an energy shortage. The concept of blockchain offers a distributed, immutable, and secure data management that can be efficiently accomplished using traditional network design. Due to the distributed locations of renewable energy resources (RESs) in MG, the blockchain-enabled P2P energy trading framework has diverse features, including immutability, traceability, auditability, verifiability, automation, and transparency [19].

### 2.1. Blockchain-Enabled P2P Energy Trading in MG

The current energy trading mechanisms experience security and scalability issues because they rely on the centralized infrastructure of a single supplier, which is a single point of failure. P2P energy transaction refers to directly exchanging a clean energy supply between individuals who can generate their own energy from their RESs [2]. An energy trading system is responsible for managing the energy assets and the market. Consequently, it must guarantee the reliability and transparency of energy flows and financial transactions across the distribution system [20]. Networked MG enables individuals to proceed P2P energy transactions to satisfy local energy demands and decrease transfer loss. The fundamental goals of energy trading are reducing energy costs, maintaining and increasing sustainable energy usage, and enhancing social connections among prosumers [13]. In recent years, there are several well-known P2P energy transaction pilot projects such as Brooklyn Microgrid [21], Olympic Peninsula GridWise [22], SolarCoin [23], NRGcoin [24], and EnergyBlockchain [25]. However, energy trading systems introduce many challenges in establishing trust among participants, market control, and protecting the participants’ sensitive information. Thus, blockchain technology is adopted to enhance anonymity, immutability, transparency, security, and trustworthiness in a decentralized manner.

**Figure 1:**
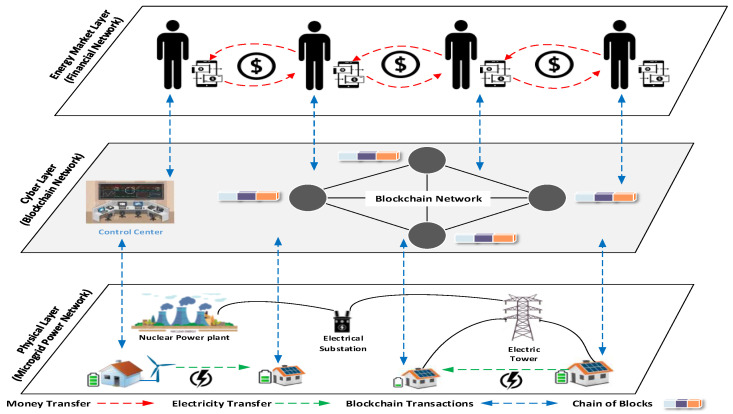
High level architecture of energy transaction in blockchain-enabled microgrid.

Figure 1 represents an example of the architecture of blockchain-enabled energy trading in microgrid. The architecture consists of three layers. The physical layer represents the physical microgrid infrastructure, including energy generation, network communication, and distribution. Houses with PV panels on top of the house or small wind turbines are considered sources of renewable energy. The generated energy from RES is consumed in the house and the surplus energy is stored in a central buffer energy storage and can be sold to neighbors who suffer from an energy shortage. The cyber layer consists of the blockchain network that is responsible for managing energy flows, data exchange, and financial transaction flows. Furthermore, it contains the energy system control center. The financial network represented in the energy market layer includes consumers and producers who bid for energy trading via the control of a cyber layer where money or digital/virtual currencies are transferred from energy buyers to sellers. The cyber layer affords a facility of participation for microgrid entities in an energy market and fosters the interaction between market and physical layers. Furthermore, it executes the energy and financial transactions created in the market layer.

### 2.2. P2P Energy Trading Design Approaches

Some recent studies have made an attempt at proposing energy trading schemes and designing energy market mechanisms using different blockchain platforms to enable P2P energy transactions. Gai et al. [2] proposed an energy trading model using consortium blockchain to mitigate linking attacks where trading information can be mined and linked with other information such as energy usage and participant location. The authors proposed account mapping that provides dummy accounts to prevent adversarial activities and conceal distribution trends. Laszka et al. [5] proposed a privacy-preserving energy transaction solution to provide secure energy trading without privacy concerns. The authors utilized blockchain technology to provide anonymity for bidding and communication. A mixing service used for user anonymity and asset workflow discussed showing how the proposed approach provides secure and privacy-preserving transactions. Kang et al. [15] proposed an energy trading model using consortium blockchain to balance local energy demand and supply. It encouraged the plug-in hybrid electric vehicles to discharge their surplus energy to gain incentives. The authors use the double auction mechanism to decide the price and energy amount for prosumers. Based on the results, the authors demonstrated that the proposed model achieves transaction security and privacy. Wang et al. [17] proposed an architecture based on a blockchain and optimization model to enable energy trading in the crowd-sourced energy system. A two-phases operation algorithm is presented to control the different operational models of crowd sources and energy peers. Aitzhan et al. [26] proposed a secure energy transaction in a smart grid based on multi-signature based blockchain and anonymous encrypted messaging streams. Zhang et al. [27] proposed a P2P secure energy trading using the Elecbay platform. The proposed work attempts to minimize the difference between local energy generation and energy demand. Energy allocation is performed using a game theory and Nash equilibrium in MG. In [28], the authors proposed an energy trading framework focused on matching energy demand and supply. A complete energy trading process was introduced where smart contracts are used to manage trading and payment rules. Paudel et al. [29] proposed a community-based energy trading using a Stackelberg non-cooperative game theory in which two separate competitions are enabled among buyers and sellers through energy trading. Zhou et al. [30] proposed a secure vehicle-to-grid energy trading framework in smart grids. The authors utilized the potentials of the integration between blockchain, edge computing, and contract theory in the P2P energy trading environment.

To design an energy trading framework, the market mechanism is an essential part that includes the allocation method which defines how the system allocates energy bids and asks. FeneChain is a blockchain-enabled energy trading system proposed in [31] to manage energy trading in industry 4.0 and mitigate cheating attacks initiated by sellers. User privacy is guaranteed by using anonymous authentication and a timed commitments-based mechanism is proposed to guarantee verifiable fairness during energy trading. Zhong et al. [32] present an energy trading auction mechanism to enable energy trading among users in different districts. The authors designed two auction mechanisms for real-time and day-ahead markets. The social maximization welfare is used to optimize energy allocation. The work in [33] proposed a distributed double auction mechanism using blockchain to enable P2P energy trading. Any peer can act as an auctioneer and the blockchain ensures its behavior. The authors proved that the distributed implementation of the double auction mechanism leads to better promotion of local energy transfer compared to the centralized auction.

Based on the related work summary in Table 1, we investigated by means of the aforementioned review that different studies are working on the idea of energy trading utilizing one of the blockchain platforms. However, most of the previous studies did not consider the protection of the sensitive information in the energy transactions and the comprehensive design of a secure energy market. In addition, most of them did not provide a performance benchmark and they assume that the performance of the blockchain is not degraded. To provide high-performance secure and privacy-preserving P2P energy trading, the enormous potentials of HF in the integration with the energy trading in the microgrid is unleashed. Benchmark analysis of the underlying HF platform and security analysis are provided to distinguish our work from the aforementioned related work papers.

## 3. Threat Model and Design Goals of P2P Energy Trading

### 3.1. Threat Model

P2P energy trading experiences various security challenges. In this paper, we focus on four types of threats, which are summarized as follows.

#### 3.1.1. Data Breaches

In order to be able to sell or buy energy, individuals have to fulfill user registration. Individuals provide personal information (ID or License number) to show their identities to the market operators for validation. Identity information could be leaked or exposed to unauthorized organizations without permission from users. Data tampering, replication, or leakage could take place if an adversary or attacker gains access to the energy market.

#### 3.1.2. Sensitive Data Leakage

Energy transactions are rich sources of sensitive information. Participants’ sensitive information could be divided into three types: personal, financial, and energy information. Personal information includes name, identification number, and location. Financial information includes credit card number or digital wallet address and account balance. Energy information includes the SM reads, the quantity of surplus or demanded energy, and the bidding price. If sensitive data leakage occurred, the unauthorized organization/attacker could reveal the identities of market participants, steal money, easily obtain how many times the participant sells or buys energy, and derive energy consumption history.

#### 3.1.3. Security and Stability Breaches of Energy Market

An attacker can modify the prices of energy assets or modify the regulatory policy in the energy trading framework. A malicious user could try to create unreliable payments or double-spend the money to cheat other users. In addition, a malicious producer may bid with a large amount of energy to sell, while he does not have the ability to produce it. A legitimate user may cooperate with other users to maximize their profits to conduct a collusion attack. A malicious user may perform a repudiation attack where the user denies the fact that they initiate a certain transaction. A buyer can buy most of the offered energy to make other buyers unable to satisfy their energy demands. This is considered a kind of denial of service (DoS) attack.

#### 3.1.4. Network Security Attacks

Compromised network entities have a serious impact on the system security that could lead to DoS, DDoS, data leakage, privilege escalation, and MITM attacks. SMs play a vital role in modern energy systems. An attacker may compromise the participant’s SM and send wrong energy measurements to the owner to deceive or destabilize the market. Eavesdropping and MITM attacks may take place during the transmission of energy transactions. Furthermore, attackers may spoof false data or tamper with the identity of a legitimate user to gain access to the energy market.

### 3.2. Design Goals

The design goals of the proposed energy trading system are summarized as follows.

#### 3.2.1. Preserving Privacy

The main goal of our proposed system is to protect sensitive information included in participants’ energy transactions. The energy trading system must ensure that adversaries are not allowed to conclude the identities of participants from their transactions nor accessing the energy transactions from the ledger. In addition, transactions should not be received or interpreted by unauthorized peers or users. SPETS must ensure data integrity to prevent data altering during the transmission from the sender to the receiver.

#### 3.2.2. Securing the Energy Market

The allocation method and market rules implemented in the market manager must be modified only by authorized users such as the network administrator. All participants must be authenticated and authorized to limit malicious behaviors and detect attackers clearly. In addition, all communications between users and the energy transaction system must be encrypted.

#### 3.2.3. Ensuring Stability of Energy Trading Market

The proposed market design must ensure preventing participants from selling or buying unreasonable amounts of energy to mitigate collusion attack, employing an efficient allocation method to increase the welfare of participants, defining the minimum and maximum price to control the energy price, and achieving reliability and trust between producers and consumers by conducting market reputation scores that change based on their behaviors.

## 4. The Proposed HF-Enabled P2P Energy Trading System

To set up a ubiquitous P2P energy trading ecosystem in MG realizing the design objectives presented in Section 3.2, a secure and privacy-preserving unified model is proposed based on the reference architecture presented in Figure 1.

### 4.1. Components of the Proposed System

- Microgrid: MG provides the physical communication between smart energy peers. MG with integrated control is responsible for providing utilities such as energy generation, energy storage, bi-directional energy transfer, and energy trading management.
- Smart Energy Sources: It is supposed that each participant has one or more renewable generation units such as PV panels or small wind turbine. In addition, participants must have SMs to monitor energy consumption and initiate and receive energy transactions to and from the blockchain.
- Participants: There are two types of participants, namely producers (sellers) and consumers (buyers). The producer is a person who has surplus energy and wants to sell it. The produced energy can be stored in local battery banks in his residence or in the energy buffer of the MG. The consumer is a person who has an energy shortage and wants to buy energy.
- Energy Management System (EMS): The EMS represented by the energy market, which is the interface between energy sellers and buyers, controls energy trading, performs energy allocation, calculates energy price, controls access to energy assets, monitors participants’ behaviors, and secures transactions. The EMS implementation is utilizing HF security, privacy, and performance features.

### 4.2. Problem Definition of Energy Trading in MG

In this subsection, the problem of energy trading among RES owners in MG is formulated. The MG management system facilitates the physical energy transactions where the proposed HF-enabled energy market is the mediator between energy sellers and buyers. It is assumed that we have a number of MG denoted as *G* and indexed by *k* where k={1,2,3,...,*k*}. There are a set of energy sellers denoted by $\overset{`}{S}=\left\{S_{i}\;|\;i\in N\right\}$ and a set of energy buyers denoted by $\overset{`}{B}=\left\{B_{j}\;|\;j\in N\right\}$. The minimum and maximum energy requirements of buyer $B_{j}$ are denoted by $B_{j}\left(R^{min}\right)$ and $B_{j}\left(R^{max}\right)$ (kWh), respectively. Similarly, the minimum and maximum energy requirements of seller $S_{i}$ are denoted by $S_{i}\left(R^{min}\right)$ and $S_{i}\left(R^{max}\right)$ (kWh), respectively. Accordingly, the total required energy (total demand) (kWh) of buyers $\overset{`}{B}$ is denoted by $D=\sum_{j=1}^{n}\psi_{j}$, from *k*. The sellers and buyers energy state is denoted by ${\mathrm{ES}}_{i}$ and ${\mathrm{ES}}_{j}$, respectively. The capacity of seller $S_{i}$ and buyer $B_{j}$ are, respectively, denoted by $S_{i}^{cap}$ and $B_{j}^{cap}$. The energy supplied by any seller $S_{i}$ is denoted by $E^{Sup}$ (kWh). Therefore, the total energy supplied (kWh) in the MGs is denoted as $E^{Tot}=\sum_{i=1}^{n}E_{i}^{sup}$, from *k*. Thence, the anticipated energy for a buyer $B_{j}$ is calculated as follows:

(1) $$E_{Anti}^{B_{j}}=\left(\psi -\eta \left(E^{Sup}\right)\right)\forall i,j\in N,and\;E^{Sup}\leq \psi$$

where η depicts the average efficiency of seller $S_{i}$. Thus, the total anticipated energy for all buyers $\overset{`}{B}$ is calculated as follows:

(2) $$\begin{array}{c} T\left(E_{Anti}\right)=\left(D-\eta \left(E^{Tot}\right)\right) \end{array}$$

The objective of the energy system is to minimize the difference between the total energy demand and the total offered energy. Based on Equations (1) and (2), the energy management problem can be formulated as follows:

(3) $$RF\left(E_{Anti}^{B_{j}}\right):\min\sum_{i=1}^{n}\sum_{j=1}^{n}\left(\psi_{j}-\eta \left(E_{i}^{Sup}\right)\right)$$

related to the following conditions:

(4) $$S_{i}\left(R^{max}\right)<S_{i}^{Cap},$$

(5) $${\mathrm{ES}}_{i}-E^{sup}\geq S_{i}\left(R^{min}\right)$$

(6) $$\psi \leq B_{j}\left(R^{max}\right),$$

where Equation (4) indicates that the capacity of $seller_{i}$ must be greater than his maximum required energy. The difference between the energy state and energy supply of $seller_{i}$ must be greater than or equal to his minimum energy requirements (Equation (5)). According to $buyer_{j}$, his maximum required energy must be less than or equal to the required energy (Equation (6)). For energy allocation, a discrete-time double auction with average mechanism is proposed to increase buyers’ and sellers’ welfare.

### 4.3. Configuration Specification of HF in SPETS

Blockchain is an emerging and fast-developing technology that was firstly introduced by Satoshi Nakamoto in 2008 [34]. Blockchain has the potential to address the security vulnerability in the internet of things (IoT) era [35]. By the end of 2015, HF was introduced by IBM and Digital Asset Holdings as one of the first projects in Hyperledger [36]. HF is a blockchain platform that implements an immutable and distributed ledger in a modular architecture that provides scalability, confidentiality, and high performance [37]. In this part, the configuration specifications of HF are examined to fulfill the design goals of the proposed energy trading system. Consequently, the main components, modules, and parameters of HF to guarantee security, authenticity, transparency, integrity, privacy, immutability, and access control are introduced.

#### 4.3.1. Identity Generation and Management

Because HF is a permissioned platform, all components, users, and entities must have digital identities (encoded in X.509 digital certificate) and cryptographic keys (public and private keys). Fabric certificate authority server (Fabric-CA) handles identity registration, digital certificates generation, and certificate revocation control. In this paper, we use the default Fabric-CA server that employs elliptic curve digital signature algorithm (ECDSA) to issue X.509 digital certificates and use the SDK to connect user application with the CA server. The Membership Service Provider (MSP) defines the trusted CA servers, validates the identities of all participants, and verifies the digital signatures of all transactions. Thenceforth, user authentication, identity management, and cryptography are provided by means of using CA and MSP.

#### 4.3.2. Channel Creation

Channel enables private communication between two or more channel members. The PDC utilized to add more privacy to sensitive data stored in the form of hashes in a side database away from unauthorized access. In this paper, a channel called *Energy_Trading* is employed as well as using two PDC definitions called *energyasset* and *energyasset_privateDetails*.

#### 4.3.3. Genesis Block Generation

The first generated block in a channel called the genesis block. The configtxgen tool is used to generate the orderer genesis block of the SOLO ordering node. After that, the channel configuration genesis block will be generated.

#### 4.3.4. HF Network Initialization

After generating the required artifacts, the endorsing and committing peers will join the pre-established *Energy_Trading* channel using the orderer node. All peer nodes have X.509 digital certificates in their configuration to determine which organization the peer node is associated with. Then, the chaincode of the energy trading is firstly installed on all endorsing peers and then instantiated on the *Energy_Trading* channel for chaincode activation.

#### 4.3.5. Secure Energy Transactions Generation

The user application is used by the sellers/buyers to invoke the chaincode of energy trading to sell/buy energy assets. The security artifacts are used to encrypt, digitally sign the transactions, and authenticate users. The transient fields in the transaction proposals are used to hide and protect the sensitive information of sellers/buyers in the energy transactions. The proposed energy market handles energy bids, allocates energy assets, and determines energy price.

## 5. The Working Mechanism of SPETS

It is essential to understand the core components and the work intuitive of SPETS. The working mechanism of the different components of the proposed system can be summarized as follows.

### 5.1. The Proposed Energy Market

The most important and powerful part of our proposed system is the design and implementation of the energy market. The proposed market divides the day into fixed-size time slots for energy trading which is represented as s∈S, where *S* represents all time slots available for trading per a day. All time slots have the same length α and each time slot has beginning time $t_{b}^{s}$ and ending time $t_{e}^{s}$ where $t_{e}^{s}=t_{b}^{s}+\alpha$. The energy sellers start to submit their asks $a_{i}^{s}\in A^{s}$ and energy buyers start to submit their bids $b_{j}^{s}\in B^{s}$ before $t_{b}^{s}$, where $A^{s}$ and $B^{s}$∈ order book $O^{s}$ in time slot *s*. The start time to submit bids and asks is $t_{sub}^{s}=t_{b}^{s}-\theta$ where θ<α. It is assumed that buyers and sellers are capable of predicting their demand/supply for any time slot *s* within the day based on their energy consumption and production profiles. In every ask ($a_{i}^{s}$), the $seller_{i}$ determines selling price per one unit ($p_{sell}^{i}$) ¢/kWh), energy source (wind or solar), and maximum energy units to be traded ($Q_{sell}^{i}$) (kWh). In every bid, the $buyer_{j}$ determines buying (bidding) price per one unit ($p_{buy}^{j}$) (¢/kWh), preferred energy source, and maximum number of energy units needed ($Q_{buy}^{j}$) (kWh).

To maintain market stability and keep the unit price in the acceptable range, $p_{sell}^{max}$ and $p_{buy}^{min}$ are denoted as maximum and minimum selling and buying prices (¢/kWh) per unit for sellers and buyers, respectively. The transaction in any time slot *s* will occur if $p_{buy}^{j}⩾p_{sell}^{i}$. Consequently, in time slot *s*, one bid $b_{j}^{s}$ can be matched with many asks $a_{i}^{s}$ from the set of asks $A^{s}$ in which unmatched bids and asks remain in the order book. If $p_{buy}^{j}$ submitted by $buyer_{j}$ is less than $p_{buy}^{min}$, the bid will be rejected by the system. Similarly, if $p_{sell}^{i}$ determined by $seller_{i}$ is greater than $p_{sell}^{max}$, the ask will be rejected by the system. Once a time slot *s* finished, the current order book will be cleared, and the new time slot will start to receive asks and bids for the next bidding process. The design of the proposed market consists of three main modules.

- Orders Manager (OM): It comprises two processes, namely orders handling and orders verification. The orders handling process is responsible for receiving orders, checking their format, and sending back the responses. If the order format is correct, it will be sent to the verification process. If not, it will be rejected, and the participant will be notified. If the process receives an order in the time before or after the submitting time, it will be buffered until the next submission time. The orders verification process examines if the orders meet the rules of the market in terms of order configuration, pricing, and participant reputation. Firstly, the order configuration includes the determination of energy source, unit price, and the number of energy units that will be produced or consumed. Secondly, the buying or selling prices must be within the price range pre-determined by the market. Finally, the reputation score of the seller must be greater than the pre-determined reputation threshold $Thr_{Rep}$. Algorithm 1 shows orders validation and verification steps. The computational complexity for this algorithm is O(1).
- The Market Manager (MM): It includes the time slot manager that is responsible for managing the time slot’s beginning and ending as well as order submitting time. All orders verified by the OM are assembled in the orders book to execute the allocation mechanism. We use a discrete-time double auction mechanism to allocate supply and demand of market participants in a specific time slot, as discussed in Algorithm 2. If there is a matching, the matching result is sent to the orders handling process to send notifications to the involved participants. The market allocation mechanism employs a maximum allocation threshold ($Thr_{Max\_Alloc}$) to mitigate the collusion attack.
- The Reputation Manager (RM): It is used to initialize and calculate the market reputation scores of sellers to guarantee market reliability and trustfulness between buyers and sellers. The reputation score initialization used to set initial scores for sellers at the beginning of their participation. By the end of time slots, reputation scores are updated by the reputation score update process and the orders verification process is notified of the scores.

**Algorithm 1** Energy Orders Handling and Verification
  1:**Input**: Seller’s ask $a_{i}^{s}$ OR buyer’s bids $b_{j}^{s}$ in time slot *s*.  2:**Output**: Orders Book $O^{s}$ contains a valid group of Asks $A^{s}$ and bids $B^{s}$ of time slot *s*.  3:Receive seller ask $a_{i}^{s}$ or buyer bid $b_{j}^{s}$  4:**if** order receiving time in correct submission time $t_{sub}^{s}$ **then**  5:      **if** Order is seller ask **then**  6:          **if** $Seller_{i}$ submit ask for the first time **then**  7:                Set initial reputation score for $Seller_{i}$  8:          **else if** Seller reputation score ≥ $Thr_{Rep}$ **then**  9:                **if** Bid price <$p_{sell}^{max}$ **then**10:                    Add seller’s ask to the order book $O^{s}$11:              **else**Reject seller’s ask12:          **else**Reject seller’s ask13:      **else if** Order is buyer bid **then**14:          **if** bid price >$p_{buy}^{min}$ **then**15:              **if** Energy quantity is valid **then**16:                   Add buyer’s bid to the order book $O^{s}$17:              **else**Reject buyer’s bid18:          **else**Reject buyer’s bid19:      **else**Unknown Order20:**else**Buffer the order until the next submission time $t_{sub}^{s+1}$21:Return orders book $O^{s}$


#### 5.1.1. Market Reputation Score (MRS)

To promote market reliability and trust between sellers and buyers, the concept of MRS is adopted. The MRS indicates the trustfulness of the seller in the energy market. The MRS is a value that started from 0 to 100, where 100 or close represents the good reputation and trust of the seller, while 0 or close represents the bad reputation and trustlessness of the seller. If $seller_{i}$ has MRS less than reputation threshold $Thr_{Rep}$, their asks will be rejected by the market because of the bad reputation. The $Thr_{Rep}$ value equals 30. With the seller’s first market participation on the day, an initial MRS in a range from 30 to 50 will be assigned to the seller. This initial MRS score will be recalculated at the end of each time slot after selling energy to the buyer according to Equation (7).

(7) $$\delta_{s}^{i}=\left\{\begin{array}{cc} \delta_{s-1}^{i}-\rho^{k}*\left({\hat{Q}}_{sell,s}^{i}-Q_{sell,s}^{i}\right), & \;\mathrm{if}\;{\hat{Q}}_{sell,s}^{i}\neq Q_{sell,s}^{i},\\ \delta_{s-1}^{i}*\left(1+\rho^{k}\right), & \mathrm{Else} \end{array}\right.$$

where $\delta_{s}^{i}$ is the RMS value in time slot *s* for seller *i*, $\rho^{k}$ is a constant weighting factor. The ${\hat{Q}}_{sell,s}^{i}$ is the offered quantity of energy units by seller *i* (kWh), $Q_{sell,s}^{i}$ is the actual quantity of energy units (kWh) generated by seller *i* in time slot *s*. The MRS calculation depends on the difference between the actually generated units and the offered units. If the difference is zero or a small value, the MRS score will increase, otherwise it will be decreased.

#### 5.1.2. Discrete-Time Double Auction

We propose a discrete-time double auction with average mechanism for efficient energy allocation and pricing. The market allocation mechanism acts as an auctioneer that obtains all bidding prices of sellers and buyers from the valid orders located in the orders book $O^{s}$ in time slot *s* and uses them to perform allocation to determine the clearing price. Algorithm 2 shows the details of the allocation mechanism. The computational complexity for Algorithm 2 is $O(N^{2}+X)$ where *N* denotes the number of energy traders in time slot *s* and *X* denotes the number of bids and asks in time slot *s*. In the discrete-time double auction, every participant submits his ask or bid price without the need to know information about other bids or asks. This approach helps us to add more privacy to the purchasing and selling operation of participants.

As mentioned before, a group of sellers $\overset{`}{S}$ and buyers $\overset{`}{B}$ intended to participate in the energy market in time slot *s*, where $\overset{`}{S}=\left\{\overset{`}{S_{1}},\overset{`}{S_{2}},\overset{`}{S_{3}},....,\overset{`}{S_{i}}\right\}\;and\;\overset{`}{B}=\left\{\overset{`}{B_{1}},\overset{`}{B_{2}},\overset{`}{B_{3}},....,\overset{`}{B_{j}}\right\}$. All selling and buying prices extracted from orders in the order book $O^{s}$ stored in $P_{sell}$ and $P_{buy}$ (¢/ kWh). First, all selling prices are sorted in an ascending order where all buying prices are sorted in a descending order. The first ask is allocated to the first bid with clearing price $p_{c}^{i,j}=\left(p_{sell}^{i}+p_{buy}^{j}\right)/2$ (¢/ kWh), if the $p_{sell}^{i}\leq p_{buy}^{j}$. If the selling quantity $Q_{sell}^{i}$ is equal to buying quantity $Q_{buy}^{j}$, the ask and the bid from the list will be deleted and the next bid and the ask will be matched. If the selling quantity $Q_{sell}^{i}$ is less than the buying quantity $Q_{buy}^{j}$, the ask $ask^{i}$ will be deleted from asks list and the buying quantity $Q_{buy}^{j}$ will be updated, as shown in step 10. If the selling quantity $Q_{sell}^{i}$ is greater than the buying quantity $Q_{buy}^{j}$, the bid $bid^{j}$ will be deleted from bids list and the selling quantity $Q_{sell}^{i}$ will be updated, as shown in step 13. Matched and unmatched asks and bids are provided at the end of the time slot *s*. The OM is responsible for informing buyers and sellers about the result of the auction.
**Algorithm 2** Discrete-Time Double Auction With Average Mechanism  1:**Input**: Valid sellers’ asks $A^{s}$ and buyers’ bids $B^{s}$∈ order book $O^{s}$ in time slot *s*.  2:**Output**: Matched asks $A^{s}$ and bids $B^{s}$ and their clearing prices P.  3:Receive valid asks and bids at the beginning of time slot *s* for period = α  4:Sort buyer’s bidding prices in descending order.  5:Sort seller’s bidding prices in ascending order.  6:**while** ($p_{sell}^{i}<=p_{buy}^{j}$) **do**  7:     Match (ask ($a^{i}$), bid ($b^{j}$))  8:     $p_{c}^{i,j}=\left(p_{sell}^{i}+p_{buy}^{j}\right)/2$  9:     **if** ($Q_{sell}^{i}<Q_{buy}^{j}$) **then**10:         $Q_{buy}^{j}\leftarrow Q_{buy}^{j}-Q_{sell}^{i}$11:         remove ask ($a^{i}$)12:     **else if** ( $Q_{sell}^{i}>Q_{buy}^{j}$) **then**13:         $Q_{sell}^{i}\leftarrow Q_{sell}^{i}-Q_{buy}^{j}$14:         remove bid ($b^{j}$)15:     **else if** $Q_{sell}^{i}=Q_{buy}^{j}$ **then**16:          remove bid ($b^{j}$)17:          remove ask ($a^{i}$)18:      Return matched bids, asks, and clearing prices

### 5.2. Participants Registration and Authentication

SM is a key component of the modern energy systems designed to measure energy consumption and provide additional data to energy suppliers in a real-time manner. If an attacker intercepts the SM reads, he can obtain the behavior of the owner, conclude how many people are in the house, occupancy hours, and the electrical devices used. Despite the great potential impact of blockchain integration with microgrids, the reliability of data generated from user devices is the main challenge [38,39]. Blockchain guarantees the immutability of data in a distributed ledger, but when blockchain receives corrupted data, they remain corrupted [40]. Therefore, in this part, we focus on validating the identity of SMs and make sure that the data are not altered during transmission. Moreover, we perform user registration and digital identity generation for all users participating in energy trading.

First, the user sends a request to obtain the identification number (serial number) of their SM. It is supposed that the connection between the user and their SM is secured by an encryption technique such as elliptic curve cryptography. The user connects to the HF CA server through the Fabric SDK. He must provide his identity information to the Fabric-CA server, for example, (national ID or driver license number and SM ID). Second, the Fabric-CA server can be configured to read from a lightweight directory access protocol (LDAP) server to authenticate the user identity. The energy supplier company stores all the identity information about customers and their SMs. Thus, there is a secure connection between the Fabric-CA server and the LDAP server of the energy supplier company. Finally, the Fabric-CA server generates an X.509 digital certificate to the user *i*$\left\{Cert_{i}\right\}$ and public and private key pair $\left\{PK_{i},SK_{i}\right\}$. The user’s digital certificate is signed with the Fabric-CA digital signature. The user uses this digital certificate to prove their identity to any participant, as long as the other users trust the certificate issuer (Fabric-CA).

### 5.3. Secure and Privacy-Preserving Energy Transactions

After successful user registration, the energy trader can participate in the energy trading market to sell/buy energy. All generated transactions are encrypted using the user’s private key and signed using the user’s digital signature. Assume $seller_{i}$ has a transaction $Tx_{n}^{i}$ where *n* is the transaction number. Then, the seller encrypts $Tx_{n}^{i}$ and adds digital signature using the seller private key $Sign_{SK_{i}}\left\{Encrypt_{\left(SK_{i}\right)}\left(Tx_{n}^{i}\right)\right\}$ and sends it on the channel. The MSP verifies the digital signature using sender’s public key $Verify_{\left(PK_{i}\right)}\left(Cert_{i}\right)$. Then, the receiver can decrypt the transaction, if they only have the public key of the sender $Decrypt_{\left(PK_{i}\right)}\left(Tx_{n}^{i}\right)$. Thus, peers in the same organization or the same consortium can decrypt the message but will not be able to alter its contents unless the sender’s private key is compromised.

#### Sensitive Data Protection in SPETS

This part discusses how HF can be utilized to protect the sensitive data of participants, prevent data leakage, hide energy consumption profiles, and protect the history of asks and bids. We mainly use:

1. HF Channels: HF channel is a way of private communication between channel members where transactions submitted to one channel are hidden from any other channel members. One channel may include different organizations, while one organization can participate in different channels. The transaction issuer must be authenticated (by means of X.509 certificate) and authorized (by MSP) to submit a transaction to the channel.
2. HF PDC: HF presents PDC to allow a group of organizations on one channel to keep private data from other organizations on the same channel. Therefore, a defined group of organizations can endorse, commit, and query private data without creating a new channel. The PDC contains private data, which is stored in a private state database called "Side-DB" that existed on peers of authorized organizations. The private data was distributed from peer-to-peer using gossip protocol instead of sending it as blocks. Furthermore, the PDC contains the hash of the private data, which is stored on all peers who joined the channel to prove the existence of the private data and used for validation and audition.

Consider a $seller_{i}$ who would like to sell surplus energy to a local $buyer_{j}$. Firstly, the SM of the $seller_{i}$ detects a surplus energy and sends an energy asset to the $seller_{i}$ to obtain permission for trading. After user acceptance, the energy asset is generated, endorsed, and stored on the ledger with a particular identification number $Asset_{id}$. The private data of the energy assets is stored in the side-DB of the authorized peers. The private data are endorsed, ordered, and stored in the form of hashes to protect data contents and used for state validation. To protect private data from unauthorized access, a JSON file called collection definition defines access to private data. In the proposed HF network, this file contains two private data collection definitions: collectionEnergyAssets and collectionEnergyAssetsPrivateDetails. To write the energy asset on the ledger, the asset is divided into two separate data definitions. The first data definition is called energyasset and the second data definition is called energyasset_PrivateDetails.

In SPETS design, all private and sensitive information about users and their energy assets are protected and privately stored in the side database of authorized peers in the form of hash values. The proposed design hides the quantity of generated energy assets, the name of the producer, the energy price, the energy source, and the production time. Thus, the production or consumption history of a participant cannot be tracked by unauthorized peers or participants.

## 6. Experiment Methodology

A permissioned HF-enabled management information system is proposed to provide P2P energy trading in MG. The key elements of the proposed HF network (Energy Network) are discussed as follows:

- Organizations: In the proposed HF network, there are three organizations, sellers, buyers, and the energy supplier, that decide to employ the proposed HF network to manage, sell, and buy energy. The sellers’ organization is responsible for handling sellers’ asks, while the buyers’ organization processes the buyers’ bids. The energy supplier organization is responsible for managing the energy network and managing users accounts and organizations.
- Peers: They host instances of chaincode and the ledger. There are two types of peers: endorsing and committing peers. Each organization consists of two peer nodes for endorsing and committing transactions. One peer is selected as an anchor peer to enable communication between the three organizations.
- Orderer: It is responsible for concurrently receiving transaction proposal responses from different users’ applications and arranging them in a well-defined sequence and combining them into blocks. Orderer sends these blocks to committing peers to commit and add them to the ledger. A single orderer node is used for implementation simplicity; it is called SOLO ordering.
- Certificate Authority: Fabric-CA is responsible for generating X.509 digital certificates to manage identities and sign transactions. The MSP defines the trusted root CA and intermediate CAs. In our energy network, the Fabric-CA is employed as root CA.
- Channel: In the energy network, there are two channels: the application channel and the system channel. The application channel, called *energy_channel*, handles the transactions coming from user applications. The other channel handles transactions of the network configuration.
- Client Application: It is the program that users can use to interact with the energy network by generating transaction proposals to invoke specific chaincode function. In our proposed system, users are classified as energy sellers or buyers.
- Chaincode: It is a program that manipulates business logic approved by the members of the network. Chaincode can be packaged, installed, instantiated, and upgraded on the endorsing nodes by the administrator or authorized user. In our energy network, chaincode handles and verifies energy bids and asks, performs energy allocation, and determines energy price. Our chaincode is implemented in go language and called Energy_Trading_SC.

**Figure 2:**
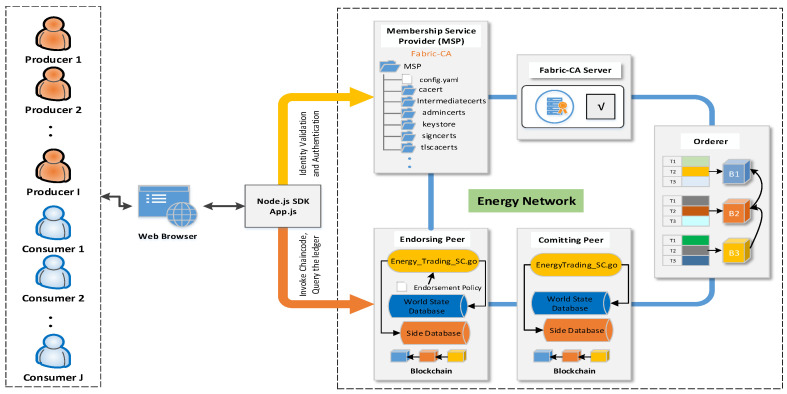
The implementation of SPETS using Hyperledger Fabric.

Figure 2 shows the implementation of the proposed energy transaction system using HF. Consumers and producers connect to the energy market using JavaScript application in their web browsers where they can register, buy, and sell energy. HF SDK for Node.js handles the communication between the application and the HF network. The information system is implemented by HF Version 1.4.0.

## 7. Security Analysis and Numerical Results

### 7.1. Security and Privacy Analysis

Security: In contrast to the traditional security and privacy protection, SPETS utilizes the private permissioned HF blockchain platform. However, SPETS inherits the security and privacy concepts of HF. We assume that an attacker has the opportunity to threaten the security of the proposed system. Table 2 summarizes eight attacks to which blockchain and energy trading systems are vulnerable. Table 2 points out how SPETS mitigates these attacks and the level of resistance against those attacks.

Privacy: In HF, it is hard for external attackers to intercept private data because only authenticated users connect to the blockchain network. The proposed implementation employs channels and PDCs to protect sensitive data using the transient field in the proposal request. The endorsement peers receive the transactions, validate them, and send the hash of data back to users in the proposal response. All sensitive data are stored in a separate database called side-DB that is visible only by authorized peers. The peer nodes use gossip protocol to deliver transactions among authorized endorsing and committing peers instead of broadcasting them. All private data are deleted from the side-DB after a configurable number of blocks. To ensure hiding the sell and buy activity of participants, the proposed market design adopts the discrete-time double auction that does not need to share bidding information between participants. In contrast, in continuous double auction, every participant needs to know all information about other participants’ bids and asks [41].

To distinguish the proposed energy trading system, our work is compared with other recently proposed energy transaction approaches, as shown in Table 3. The table shows the drawbacks of other proposed approaches. Therefore, SPETS overcomes the weakness points by addressing the missed features, such as sensitive information protection, designing an entire energy system, securing the energy market, adopting participants’ reputations, and performing benchmark analysis.

### 7.2. Case Study

This section includes the experimental setup, the included energy trading scenarios, the experimental results and analysis, and the benchmark analysis of the proposed energy network.

#### 7.2.1. **Simulation Setup**

The experiments are conducted using HF Version 1.4.0 [37], run on personal computer with Intel(R) core i5-7400 CPU @ 3.00 GHZ, 8GB memory, and Ubuntu 16.04.4 TLS. Docker containerization is used to build our HF energy network. In this case study, two scenarios are considered to demonstrate the effectiveness of the proposed energy market.

- The first scenario is the base line in our experiment where all participants are connected to the grid without applying any market mechanism. Feed-in Tariff (FIT) is used when producers sell their surplus energy to the grid and Time of Use Tariff (ToU) is used when energy is transferred from the grid to consumers.
- In the second scenario, producers and consumers participate in the energy market as sellers and buyers to trade energy following the proposed energy trading system.

**Figure 3:**
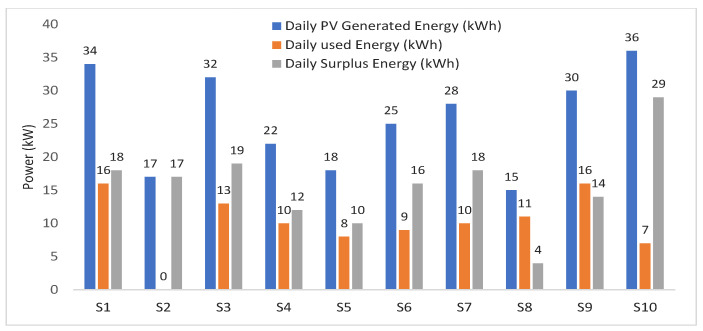
Sellers’ PVs generated, consumed, and surplus energy throughout one day.

In our case, there are 20 participants grouped as 10 sellers and 10 buyers. For simplicity, it is assumed that all producers have PV cells and the experiments executed for one time slot of energy trading. The solar system energy output is estimated for all producers using the PVWatts® Calculator web application, developed by the National Renewable Energy Laboratory (NREL) [47]. This free tool is a powerful tool to help users estimate the production of solar panels without scanning the home roof. However, it depends on the data provided from 200 solar installers throughout the USA and the pricing tables of each utility. The location of the solar panel systems selected is in Los Angeles, USA, with latitude and longitude equal to 34.05–118.26, and the time is July. The tilt is set to 20 degrees, azimuth is set to 180 degrees, and system loss is 9.59%. The solar panel systems energy output of all producers, the daily use, and the surplus energy are shown in Figure 3. Sellers consume part of the PV generated energy in their homes and the surplus energy will be sold. As shown in Figure 3, $seller_{10}$ has the highest surplus energy (29 kWh) where the lowest surplus energy is produced by $seller_{8}$ (4 kWh). The total energy demand required for $buyer_{1}$ to $buyer_{10}$ are 15, 9, 15, 14, 18, 7, 11, 8, 16, 22 (kWh), respectively.

In the experiments, the time slot α = 1 h, the time to receive bids and asks θ = 15 min from the beginning of the time slot. Based on the reported average pricing of energy in Los Angeles in 2020, which is 19 (¢/kWh) [48], the maximum bidding price for sellers $p_{sell}^{max}$ = 25.00 (¢/kWh) and the minimum bidding price for buyers $p_{buy}^{min}$ = 15.00 (¢/kWh). As previously mentioned, the initial values of the seller’s MRS = {32, 38, 45, 34, 40, 45, 50, 42, 44, 36} that will assigned with the first participation on the day. These values are updated using Equation (7), after completing energy allocation in every time slot *s*. The reputation threshold $Thr_{Rep}$ = 30, the weighting factor $\rho^{k}$ = 0.25. $Thr_{Max\_Alloc}$ calculated as 25% of the total offered energy in the time slot, which equals 39.25 (kWh). In the first scenario, the FIT and ToU equal 17 and 22 (¢/kWh), respectively.

#### 7.2.2. **Result Analysis**

This section provides a numerical analysis to demonstrate the efficiency of the proposed energy market in terms of energy allocation and reputation methods.

In the second scenario, all valid asks, bids, and bidding prices in the time slot *s* are shown in Table 4. The discrete-time double auction explained in Algorithm 1 is applied to allocate energy and determine clearing price of matched asks/bids. The results of the allocation are shown in Table 5, where $Q_{i}^{j}$ determines the energy quantity (kWh) of transferred energy from $seller_{i}$ to $buyer_{j}$ and $p_{c}^{i,j}$ is the clearing price of transferred energy (¢/kWh) from $seller_{i}$ to $buyer_{j}$.

**Figure 4:**
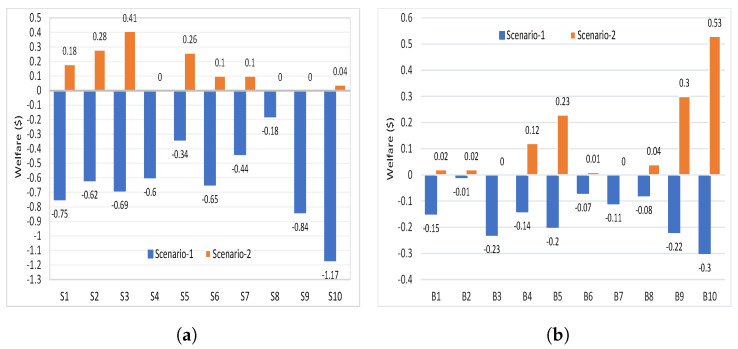
2 Welfare values of scenario-1 and scenario-2. (**a**) Welfares of energy sellers; (**b**) Welfares of energy buyers.

The welfare of all sellers in scenario-1 and scenario-2 is shown in Figure 4a, which is calculated using Equation (4). The welfare of sellers is calculated as the difference between the clearing price of transferred energy (¢/kWh) from $seller_{i}$ to $buyer_{j}$ and the bidding price of $seller_{i}$ multiplied with the quantity of energy in (kWh) transferred from $seller_{i}$ to $buyer_{j}$. The welfare of all buyers is shown in Figure 4b, which is calculated using Equation (9). Similarly, the welfare of buyers is calculated as the difference between the bidding price of $buyer_{j}$ and the clearing price of transferred energy (¢/kWh) from $seller_{i}$ to $buyer_{j}$ multiplied with the quantity of energy in (kWh) transferred from $seller_{i}$ to $buyer_{j}$.

(8) $$W_{seller(i)}=\left(p_{sell}^{i}-p_{c}^{i,j}\right)*Q_{i}^{j}$$

(9) $$W_{Buyer(j)}=\left(p_{c}^{i,j}-p_{buy}^{j}\right)*Q_{i}^{j}$$

It can be seen that the welfare of $seller_{4},seller_{8},$ and $seller_{9}$ is zero, where they did not sell energy to buyers because they bid with high prices. Similarly, $buyer_{3}$ did not buy energy and his welfare is zero because he bid with the lowest price. The welfare of $buyer_{7}$ is zero because the bidding price is equal to clearing price ($p_{sell}^{7}=p_{c}^{7,7}=21.00\left(¢/kWh\right)$). Thus, in the following time slot, $seller_{4},seller_{8},$ and $seller_{9}$ will try to reduce their energy prices so that they can sell it, which helps reduce the price of energy. In scenario-2, all other buyers and sellers have positive welfare. In scenario-1, buyers and sellers have negative welfare because the price of energy for buyers in scenario-1 is higher than scenario-2, and for sellers it is cheaper.

**Figure 5:**
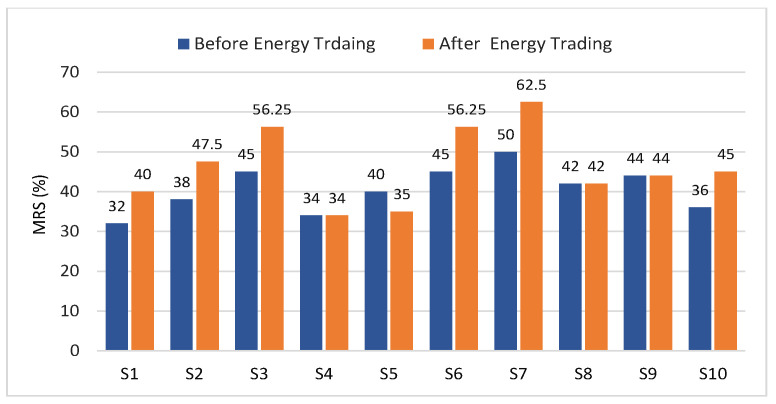
MRS of all sellers after trading in time slot *s*.

The reputation scores of all sellers are shown in Figure 5, where the reputation scores of $seller_{1,2,3,6,7,10}$ are increased because the actually produced energy is equal to the offered energy in their asks. Since $seller_{4,8,9}$ did not sell energy in this time slot, the MRS remains the same. The reputation score of $seller_{5}$ decreased because the actual transferred energy is 5 kWh where the offered energy is 10 (kWh). If the reputation score becomes lower than 30%, the seller will be banned until the end of the day.

### 7.3. Benchmark Analysis of the Proposed HF Network

Hyperledger caliper is one of the tools developed under the Hyperledger project to enable blockchain benchmarking [49]. It allows blockchain designers to measure the performance of their designed networks in different scenarios and use cases. In simpler words, the caliper tool can be viewed as a service in which we can evaluate a workflow based on a pre-determined system under test (SUT). In order to evaluate the proposed HF network under different workloads, Table 6 shows different workloads used in our experiments.

**Figure 6:**
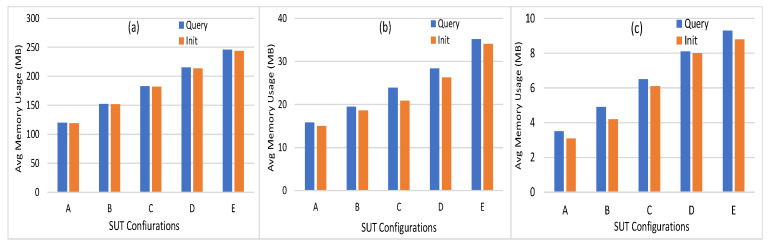
Average memory usage of (**a**) peer nodes, (**b**) orderer node, (**c**) chaincode.

**Figure 7:**
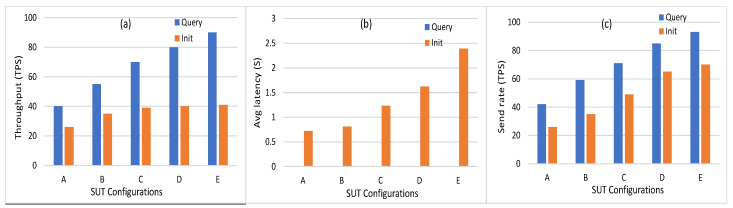
(**a**) Throughput. (**b**) Average latency. (**c**) Send rate for the five SUT configurations in HF 1.4.0.

**Figure 8:**
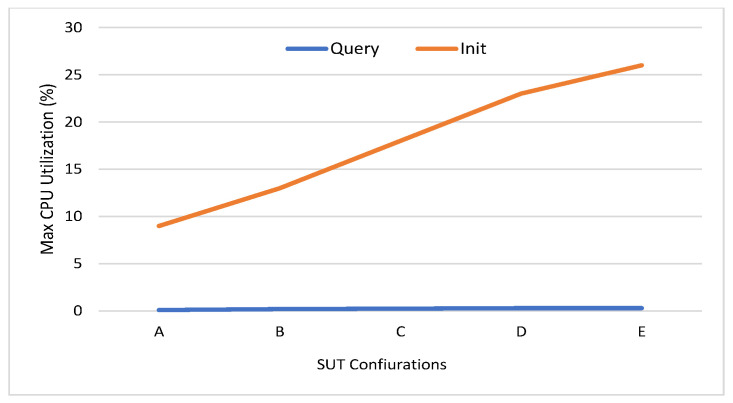
Maximum CPU usage of endorsing peer nodes.

Among different monitors in caliper, the docker monitor is utilized to monitor and evaluate the containerized peers, chaincode, and organizations on the hosted machine. The different benchmark metrics can be summarized as follows:

- Memory Usage: This metric measures the maximum and average memory consumption for every node and chaincode. Figure 6 shows the average memory usage of peer nodes, orderer node, and chaincode. It can be seen that the average memory consumption of the peer node is low, where it starts with 129 MB in workload A to reach 245.9 MB in workload E. In addition, the orderer and the chaincode consume a very low memory level (35.2 and 9.3 MB, respectively) in workload E. The minimum level of memory is consumed by the chaincode (from 3.5 MB to 9.3 MB). This indicates that the implementation of the proposed energy network can be easily implemented on devices with limited capabilities, such as smart IoT devices.
- Disk Usage: It is used to evaluate the ledger performance in terms of size and determine whether or not the ledge needs pruning. During the invoke transactions, the HF does not have to make a read on the ledger. Similarly, during the query transaction, HF reads data from the world state database. Thus, invoke and query transactions are providing zero bytes on disk. All peer nodes are writing an equal number of bytes on the disk with a maximum of 8.5 MB. The orderer node writes a number of bytes equal to 15.3 MB on the disk.
- Traffic: The traffic In and traffic Out are the parameters employed to conduct the network utilization analysis. The convergence of this study with others will provide highly intuitive and detailed guidance on performance. The traffic In and Out values for orderer peer are 4.7 MB and 8.6 MB, respectively. The peer nodes traffic In is in the range of 0.65 MB to 5.7 MB, where the traffic Out is in the range 0.32 MB to 3.6 MB. The CA traffic In and Out values are 152 bytes and zero bytes, respectively.
- Performance Metrics: Transaction throughput, latency, and send rate are the metrics that represent the HF platform’s efficiency. Figure 7 shows the number of transactions per second for different SUTs where it is in the range (40 TPS to 90 TPS) in the query and (26 TPS to 41 TPS) in the Init transactions. The average latency of query transactions is approximately zero where the Init transactions consume average latency from between 0.72 s and 2.39 s. The send rate for Init transactions is in the range (26 TPS to 70 TPS) where the query transactions rate is in the range (42 TPS to 93 TPS).
- CPU Utilization: This metric measures the CPU utilization of different peer nodes that help to analyze the chaincode operations using CPU utilization. Furthermore, it can be used to detect abnormal behavior of the HF network entities. Figure 8 shows the CPU utilization of endorsing peer nodes, which is in the range of (9% to 26%) in Init transactions, while it nears zero in the query transaction. The average and maximum CPU utilization of the chaincode and CA is approximately zero because the ordering service and the certificate generation are not run at the same time as the transaction proposal processing. However, the orderer node consumes a maximum of 15% of the CPU utilization because one orderer node is used (SOLO ordering).

Based on the result of benchmark analysis, the proposed energy trading system achieved high performance, system scalability, and consumed minimum resources that reflect the efficient design and implementation of the proposed system. Moreover, the security and privacy analysis proves the superiority of our system over several attacks.

## 8. Conclusions and Future Work

In this paper, a secure and privacy-preserving energy trading system is proposed for P2P energy trading in microgrid. In the proposed system, the essential security requirements are inherited from the HF platform to secure the energy transactions between energy sellers and buyers. Moreover, the HF channels and PDC are converged to provide fine-grained transactions privacy that protects the private information of users included in the energy transactions. The energy allocation and pricing are carried out using an efficient lightweight discrete-time double auction mechanism where the welfare of energy buyers and sellers is increased by approximately 26% and 76.5%, respectively. The proposed energy market is secured against attacks and misbehavior by malicious users, whereas the market reputation score method is conducted to ensure market reliability and trust between untrusted users. Moreover, in order to ensure the integrity of smart meter reads and guarantee users’ identification, all users are authenticated and authorized to sell or buy energy. The security analysis demonstrated that the proposed system is secure against several attacks, such as MITM, appending, linking, device injection, and collusion attacks. In order to ensure system performance and scalability, a benchmark analysis was executed. The benchmark results demonstrated that the proposed system consumed minimum resources with a maximum of 245.9 MB of memory, 8.5 MB of disk space, and 4.7 MB and 8.6 MB of In/Out traffic. Furthermore, it had a very small transaction latency of 2.39 seconds and high throughput of 90 TPS. Therefore, the proposed system using HF can be implemented in different energy applications such as smart grids, internet of vehicles, electric vehicle charging, etc. The limitations of the proposed energy trading system are that it did not address the energy trading between persons located in different microgrids. Moreover, the details of the payment method are not considered. In future work, we plan to extend our work to address these limitations and employ zero-knowledge proof (ZKP) to achieve authentication anonymity, increase transaction privacy, and mitigate transaction linkability. In addition, formulating the energy trading problem as a satisfaction function and integrating the proposed energy trading system in a different environment such as electric vehicle charging.

## Figures and Tables

**Table 1:** Summary of P2P energy trading approaches in the related work.

Reference	Parameters					
	Scenario	Market Design	Security Analysis	Privacy Consideration	Infrastructure Management	Performance Analysis
[2]	Smart Grid	⊗	⊗	*√*	CoCB	⊙
[5]	Microgrid	⊙	*√*	*√*	HF	⊗
[15]	Electric vehicles	⊙	*√*	*√*	CoBC	⊙
[17]	Smart Grid	⊙	⊗	⊗	HF	⊙
[26]	Smart Grid	*√*	*√*	*√*	PBC	⊙
[27]	Microgrid	⊙	⊗	⊗	GT	⊙
[28]	Microgrid	*√*	⊗	⊗	Eth	⊙
[29]	Microgrid	*√*	⊗	⊗	GT	⊙
[30]	V2G	⊙	*√*	⊗	CoBC	⊙
[31]	IIoT	⊙	*√*	⊙	CoBC	⊙
[32]	Multi Energy System	⊙	⊗	⊗	Auction	⊙
[33]	Smart Grid	⊙	⊗	⊗	distributed Auction	⊙
Proposed Work	Microgrid	*√*	*√*	*√*	HF	*√*

*√* considered, ⊙ partially considered, ⊗ not considered, IIoT industrial internet of things, V2G vehicle-to-grid, CoBC consortium blockchain, HF Hyperledger Fabric, GT game theory, Eth Ethereum, PBC public blockchain.

**Table 2:** Possible attacks on blockchain and energy trading markets.

Attacks	Definition	Defense	Resistant to Attack
Appending Attack	Attacker compromises a peer node to generate blocks with fake transactions.	The MSP manages the identity of all peer nodes using digital signatures generated from trusted certificate authority and defines permissions of nodes over network resources.	High.
Device Injection Attack	Malicious user tries to join the energy trading system with fake or compromised SM to provide incorrect transactions to cheat other users and make the system unstable.	SPETS validates the identity of the SM from the DSO company database and ensures the data that comes from the SM is encrypted and not altered.	High.
Linking Attack	Attacker uses the information in the transactions to link data in the ledger with the same ID to conclude the energy consumption history, leak user sensitive data, or reveal the identity of a participant.	SPETS uses the channels and private data collection to make hashes of the private data of participants and store it in a side-DB that is only accessible for authorized nodes from authorized organizations.	Beyond High.
Distributed DoS (DDoS) Attack.	Attacker tries to make a service unavailable to users by sending a huge number of requests to overwhelm the HF peers and becomes unable to handle them. Attackers flood the endorsing peers with a huge number of transactions to overwhelm the network.	HF mitigates DDoS attacks using redundant architecture such as using Raft and Kafka instead of single orderer. Separating transaction execution functionalities in HF helps to increase efficiency and accelerate delivery of transactions to ordering services. Determining fixed time slots for receiving traders’ bids/asks reduced the number of transactions possibly received by the endorsing peers.	Beyond High.
Man-in-the-Middle (MITM) Attack	An adversary intercepting user data to control the communication channel between the seller/buyer and the energy market. Then, the adversary can hijack the network stream and tamper with data transmitted on the compromised channel	HF secures communication between peers using transport layer security (TLS), where any peer can work as a TLS client or server. Furthermore, hashing algorithms such as SHA-256 are used to ensure data integrity and authentication.	High.
Collusion Attack	Prosumers can collude with others to maximize their profit.	SPETS determines a maximum allocation of energy per time slot for participants to mitigate a collusion attack. Moreover, prosumers and consumers can participate once every time slot.	Beyond High.
First Preimage Attack	The attacker obtains the victim’s original seed and performs a collision search using an elliptic curve to find a seed message that gives the same hash as the victim’s private key.	All transactions are encrypted and signed using private key, and hence only the receiver could decrypt the transaction. Consequently, a minor change in any transaction will break the integrity and signature of the transaction	High.
51% Attack	If the attacker can control more than 51% of peer nodes in the network, the 51% attack can occur. The controlling nodes can prevent new transactions being confirmed and help accept fake blocks.	The modular architecture of HF and severity of consensus protocols make it suitable for different business needs. The 51% attack is very unlikely to happen if the HF network is correctly set up and configured.	Moderate.

**Table 3:** Comparison between recent works in energy trading and our work.

Ref.	Year	Paper Summary	Scope						
			Utilized Blockchain	Energy Trading Framework	Benchmark Analysis	Application Designing	System Security	Sensitive Data Protection	Market Designing
[5]	2017	Energy transactions enabled in a privacy-preserving manner in microgrid.	HF	⊙	⊗	⊗	*√*	*√*	⊙
[17]	2019	HF-enabled energy trading for crowd- sourced energy systems in smart grids.	HF	⊙	⊗	*√*	⊗	⊗	⊙
[42]	2018	Hyperledger-enabled emission trading system to provide credible trading service for legal polluters.	HF	⊙	⊙	*√*	⊗	⊗	⊙
[43]	2020	Reliable and scalable surplus energy trading among neighbors using HF.	HF	⊙	⊗	⊗	⊗	⊗	⊙
[44]	2020	Adopting smart contract to control energy bidding process to enable dynamic pricing of energy trading in smart cities.	HF	⊙	⊗	*√*	⊙	⊗	⊙
[45]	2020	P2P energy management system based on permissioned blockchain with privacy protection.	HF	⊙	⊗	*√*	*√*	*√*	⊙
[46]	2021	Secure energy transaction based on distributed pricing, scheduling, and theft mitigation.	HF	⊙	⊗	⊗	*√*	⊗	*√*
Proposed work		Utilizing HF to implement energy trading system with secure energy market, sensitive information protection, reputation management, and allocation control.	HF	*√*	*√*	*√*	*√*	*√*	*√*

*√* considered, ⊙ partially considered, ⊗ not considered, HF Hyperledger Fabric.

**Table 4:** Details of asks and bids in time slot *s*.

Sellers’ Asks			Buyers’ Bids		
**Seller** **No.**	$Q_{\mathrm{sell}}^{i}$ **(kWh)**	$p_{\mathrm{sell}}^{i}$ **(¢/kWh)**	**Buyer** **No.**	$Q_{\mathrm{buy}}^{j}$ **(kWh)**	$p_{\mathrm{buy}}^{j}$ **(¢/kWh)**
1	18	20.20	1	15	21.10
2	17	19.00	2	9	21.30
3	19	18.50	3	15	19.50
4	12	22.00	4	14	22.00
5	10	17.90	5	18	22.25
6	16	20.50	6	7	21.20
7	18	21.00	7	11	21.00
8	4	21.50	8	8	21.50
9	14	23.00	9	16	22.50
10	29	20.90	10	22	23.00

**Table 5:** Energy allocation and clearing prices in time slot *s*.

	${\mathrm{Buyer}}_{1}$		${\mathrm{Buyer}}_{2}$		${\mathrm{Buyer}}_{3}$		${\mathrm{Buyer}}_{4}$		${\mathrm{Buyer}}_{5}$		${\mathrm{Buyer}}_{6}$		${\mathrm{Buyer}}_{7}$		${\mathrm{Buyer}}_{8}$		${\mathrm{Buyer}}_{9}$		${\mathrm{Buyer}}_{10}$	
	$Q_{i}^{1}$	$p_{c}^{i,1}$	$Q_{i}^{2}$	$p_{c}^{i,2}$	$Q_{i}^{3}$	$p_{c}^{i,3}$	$Q_{i}^{4}$	$p_{c}^{i,4}$	$Q_{i}^{5}$	$p_{c}^{i,5}$	$Q_{i}^{6}$	$p_{c}^{i,6}$	$Q_{i}^{7}$	$p_{c}^{i,7}$	$Q_{i}^{8}$	$p_{c}^{i,8}$	$Q_{i}^{9}$	$p_{c}^{i,9}$	$Q_{i}^{10}$	$p_{c}^{i,10}$
$Seller_{1}$	-	-	-	-	-	-	8	21.10	10	21.23	-	-	-	-	-	-	-	-	-	-
$Seller_{2}$	-	-	-	-	-	-	-	-	8	20.62	-	-	-	-	-	-	9	20.75	-	-
$Seller_{3}$	-	-	-	-	-	-	-	-	-	-	-	-	-	-	-	-	7	20.50	12	20.75
$Seller_{4}$	-	-	-	-	-	-	-	-	-	-	-	-	-	-	-	-	-	-	-	-
$Seller_{5}$	-	-	-	-	-	-	-	-	-	-	-	-	-	-	-	-	-	-	10	20.45
$Seller_{6}$	-	-	2	20.90	-	-	6	21.25	-	-	-	-	-	-	8	21.00	-	-	-	-
$Seller_{7}$	-	-	-	-	-	-	-	-	-	-		-	11	21.00	-	-	-	-	-	-
$Seller_{8}$	-	-	-	-	-	-	-	-	-	-	-	-	-	-	-	-	-	-	-	-
$Seller_{9}$	-	-	-	-	-	-	-	-	-	-	-	-	-	-	-	-	-	-	-	-
$Seller_{10}$	15	21.00	7	21.10	-	-	-	-	-	-	7	21.05	-	-	-	-	-	-	-	-

**Table 6:** System under test performance attributes in our experiments.

Attributes		Number of Clients	Number of Transactions	Transaction Arrival Rate	Batch Timeout (ms)	Max Message Count
SUT	A	10	100	60	200	50
	B	20	200	120	350	100
	C	30	300	180	500	150
	D	40	400	240	650	200
	E	50	500	300	700	250

## Data Availability

Data is contained within the article.

## Appendix A

Table A1 and Table A2 summarize the notations and acronyms used in our work.

**Table A1:** Notations used in this paper.

Acronym	Description
*G*	Set of Microgrids
$\overset{`}{S}$	Set of Energy Sellers
$\overset{`}{B}$	Set of Energy Buyers
$S_{i}$	Energy Seller
$B_{j}$	Energy Buyer
$B_{j}\left(R^{min}\right)$	Minimum Energy Requirement for $B_{j}$
$S_{i}\left(R^{min}\right)$	Minimum Energy Requirement for $S_{i}$
$B_{j}\left(R^{max}\right)$	Maximum Energy Requirement for $B_{j}$
$S_{i}\left(R^{max}\right)$	Maximum Energy Requirement for $S_{i}$
D	Total Energy Demand in MG
${\mathrm{ES}}_{i}$	Energy State of $S_{i}$
${\mathrm{ES}}_{j}$	Energy State of $B_{j}$
$E^{Sup}$	Supplied Energy by $S_{i}$
$E^{Tot}$	Total Supplied Energy by $\overset{`}{S}$
$E_{Anti}^{B_{j}}$	Anticipated Energy of $B_{j}$
$T(E_{Anti})$	Total Anticipated Energy of $\overset{`}{B}$
s	Slot Time
$t_{b}^{s}$	Slot Time Beginning
$t_{e}^{s}$	Slot time Ending
$a_{i}^{s}$	Ask of $S_{i}$
$b_{j}^{s}$	Bid of $B_{j}$
$t_{sub}^{s}$	Submission Time of Asks and Bids
$p_{sell}^{i}$	Selling Price of $S_{i}$
$Q_{sell}^{i}$	Maximum Energy Units to be Trade by $S_{i}$
$p_{buy}^{j}$	Purchasing Price of $B_{j}$
$Q_{buy}^{j}$	Maximum Energy Units Required by $B_{j}$
$p_{sell}^{max}$	Maximum Energy Selling Price
$p_{buy}^{min}$	Minimum Energy Purchasing Price
$P_{sell}$	Total Energy Sell Prices
$P_{buy}$	Total Energy Purchase Prices
$O^{s}$	Orders Book in Time Slot *s*
$Thr_{Rep}$	Reputation Threshold of Energy Sellers
$Thr_{Max\_Alloc}$	Maximum Energy Allocation Threshold
$\delta_{s}^{i}$	RMS Value in Time Slot *s*
${\hat{Q}}_{sell,s}^{i}$	Offered Quantity of Energy Units by $S_{i}$
$Q_{sell,s}^{i}$	Actual Quantity of Energy Units Generated by $S_{i}$
$Cert_{i}$	Digital Certificate of $User_{i}$
$\left\{PK_{i},SK_{i}\right\}$	Public and Private Key Pair
$Tx_{n}$	Transaction Number n
$p_{c}^{i,j}$	Clearing price of transferred Energy from $S_{i}$ to $B_{j}$
$W_{seller(i)}$	Welfare of $S_{i}$
$W_{Buyer(j)}$	Welfare of $B_{j}$

**Table A2:** Acronyms used in this paper.

Acronym	Definition
CA	Certificate Authority
CoCB	Consortium Blockchain
DER	Distributed Energy Resources
DDoS	Distributed Denial of Service
DoS	Denial of Service
ECDSA	Elliptic Curve Digital Signature Algorithm
EMS	Energy Management System
FIT	Feed-in Tariff
GW	Gigawatt
HF	Hyperledger Fabric
MG	Microgrid
MITM	Man in the Middle
MM	Market Manager
MRS	Market Reputation Score
MSP	Membership Service Provider
NREL	National Renewable Energy Laboratory
P2P	Peer-to-Peer
PDC	Private Data Collections
PM	Orders Manager
PV	Photovoltaic
RES	Renewable Energy Resources
RM	Reputation Manager
SM	Smart Meter
SPETS	Secure and Privacy-Preserving Energy Trading System
SUT	System Under Test
TLS	Transport layer Security
ToU	Time of Use Tariff
TPS	Transactions Per Second
